# Evaluating a program to prevent anxiety in children of anxious parents: a randomized controlled trial

**DOI:** 10.1111/jcpp.14151

**Published:** 2025-03-12

**Authors:** Sigrid Elfström, Anna Rosengren, Rebecca Andersson, Johanna Engelbrektsson, Albin Isaksson, Micaela Meregalli, Livia van Leuven, Maria Lalouni, Lars‐Göran Öst, Ata Ghaderi, Johan Åhlén

**Affiliations:** ^1^ Department of Global Public health Karolinska Institutet Stockholm Sweden; ^2^ Department of Clinical Neuroscience Karolinska Institutet Stockholm Sweden; ^3^ Health Care Services Stockholm County Stockholm Sweden; ^4^ Department of Psychology Stockholm University Stockholm Sweden

**Keywords:** Anxiety disorders, prevention, parenting, internet‐based intervention, randomized controlled trial, public health

## Abstract

**Background:**

Pediatric anxiety disorders are prevalent, particularly among children with anxious parents. This trial evaluated a program for anxious parents aimed at preventing offspring anxiety disorders and symptoms over 12 months.

**Methods:**

This parallel, randomized, controlled, open‐label trial was conducted at Karolinska Institutet, Stockholm, Sweden. Inclusion criteria comprised heightened parental anxiety and the child (5–9 years old) not currently meeting criteria for an anxiety disorder. The program, Confident Parents–Brave Children (CPBC) involves six video conferencing group sessions. An external researcher randomly allocated (1:1) participants to CPBC or a self‐help control. The primary outcome was change in clinical severity ratings (CSR) between pre‐ and 12‐month assessments, assessed by the Anxiety Disorders Interview Schedule. Secondary outcomes included parent‐rated child anxiety symptoms and parental self‐efficacy. The study was preregistered at ClinicalTrials.gov (NCT04722731).

**Results:**

The trial included 215 parents (91% female) and 277 children (48% girls, mean age 7.0). At the 12‐month assessment, no statistically significant difference was found between conditions on the primary outcome (change in CSR), OR = 0.67 (95% CI: 0.30, 1.48). No statistically significantly lower prevalence of anxiety disorder at the 12‐month assessment was found in the CPBC group compared with the control group, OR = 0.57 (95% CI: 0.24, 1.31). When stratifying by age, children 5–6 years in CPBC showed lower risk of increased CSR, OR = 0.24 (95% CI: 0.08, 0.77), and anxiety diagnosis, OR = 0.23 (95% CI: 0.05, 0.84), compared to controls. Regarding secondary outcomes, CPBC children exhibited larger decreases in anxiety symptoms than control children from pre‐ to the 12‐month assessment, Cohen's *d* = .35 (95% CI: 0.15, 0.55). Parents in both conditions showed increased parental self‐efficacy over time, with no significant between‐group effect. The 12‐month assessment was completed by 204 parents (95%).

**Conclusions:**

The CPBC may have potential for preventing anxiety in young children; however, further research is warranted.

## Introduction

Anxiety disorders are the most prevalent pediatric psychiatric conditions, with children of anxious parents being at a particularly high risk (Polanczyk, Salum, Sugaya, Caye, & Rohde, 2015; Uher et al., 2023). Twin studies attribute the familial pattern of anxiety partly to genetics (Cheesman, Rayner, & Eley, 2019). Shared environmental effects on child anxiety have also been observed, although findings have been inconsistent (Cheesman et al., 2019; Knopik, Neiderhiser, DeFries, & Plomin, 2017). Further, ‘children of twins’ and adoption studies support environmental transmission of anxiety from parent to child (Eley et al., 2015; Kendler, Abrahamsson, Ohlsson, & Sundquist, 2022). Theoretical models emphasize the role of parenting in the development of child anxiety, focusing on three key dimensions. First, *overprotection* may hinder children's independence and mastery of challenging situations, leading to increased anxiety (Chorpita & Barlow, 1998; McLeod, Wood, & Weisz, 2007). Second, *anxious modeling* occurs when parents display anxiety‐related behaviors in the presence of their children, such as avoiding feared situations, displaying tension, or verbalizing anxious thoughts (Drake & Ginsburg, 2012). Through observational learning, children may adopt similar anxious patterns in behavior, feelings, and thinking. Third, parental criticism or lack of warmth has been theorized to increase the risk of childhood anxiety, by giving the child a low sense of self‐worth and competence, and making the child see the surrounding world as hostile (Drake & Ginsburg, 2012). Empirical studies support the association between child anxiety symptoms and parental anxious modeling and overprotection (Askew & Field, 2008; Lawrence, Waite, & Creswell, 2019). However, findings on the roles of parental criticism and warmth remain inconsistent (McLeod et al., 2007).

Interestingly, parent‐only interventions have demonstrated effectiveness in treating pediatric anxiety disorders in several randomized controlled trials (RCT; Jewell, Wittkowski, & Pratt, 2022). Given the familial component of anxiety and the effectiveness of parent‐based treatments, targeting anxious parents has emerged as a promising prevention strategy. So far, only one full‐scale RCT (Ginsburg, Drake, Tein, Teetsel, & Riddle, 2015) investigating the prevention of anxiety disorders in children of anxious parents has been conducted as a true prevention study (i.e. including only children with no current anxiety disorder, and utilizing the occurrence of anxiety disorders as the outcome). In the study, 136 families were randomized to an 11‐session family intervention, the Coping and Promoting Strength Program (CAPS), or a control condition receiving an anxiety information pamphlet. At the 12‐month assessment, children in the intervention group had a 5% incidence of anxiety disorders, compared to 31% in control children. Although the CAPS demonstrated positive outcomes, its feasibility as a preventive intervention is questionable, given the number of therapist hours required per family. Besides being financially costly, families with less‐anxious children may be reluctant to engage in an intensive program requiring active participation from both children and parents.

Following Ginsburg's study, Åhlén, Vigerland, Lindberg, Gunterberg, and Ghaderi (2022) conducted a pilot study (*N* = 40) evaluating a less extensive parent‐only intervention, the Strengthening Anxious Parents Program (SAPP). Based on the pilot results, a new parenting group program adapted for video conferencing was developed: Confident Parents–Brave Children (CPBC). Alongside preventing child anxiety, CPBC aims to increase parental self‐efficacy, which is defined as a parent's confidence in executing effective child‐rearing behaviors (Ardelt & Eccles, 2001).

The aim of this study was to evaluate the efficacy of the CPBC program by addressing the following research questions:

1. Is the CPBC program effective in preventing childhood anxiety disorders within a period of 12 months, compared to a self‐help parenting book?
2. Is the effect moderated by the severity of parent anxiety or child anxiety symptoms at baseline, or child gender or age?
3. Is the CPBC program effective in preventing childhood anxiety symptoms within a period of 12 months, compared to a self‐help parenting book?
4. Is the CPBC program effective in increasing parental self‐efficacy within a period of 12 months, compared to a self‐help parenting book?

## Method

### Study design

The current parallel, randomized, controlled, open‐label trial was conducted at Karolinska Instituted, Stockholm, Sweden. Assessments were conducted at baseline, postintervention, and 12‐month after inclusion. The primary outcome was change in clinical severity ratings (CSR) between pre‐ and 12‐month assessments, assessed by the Anxiety Disorders Interview Schedule for Children (ADIS‐C; Silverman & Albano 1996). The study was approved by the Swedish Ethical Review Authority (2020‐03532), followed the Consolidated Standards of Reporting Trials (CONSORT) guidelines, and was preregistered at clinicaltrails.gov (Prevention of Childhood Anxiety Disorders in Offspring of Anxious Parents; https://classic.clinicaltrials.gov/ct2/show/NCT04722731). This paper addresses preregistered research questions one to four, while questions five and six (regarding mediators of change and cost‐effectiveness) and 36‐month follow‐up results will be covered in future publications.

### Participants

The trial was advertised in a regional newspaper and on Facebook. Parents who clicked the advertisement were directed to a website with study information and a link to a screening survey. Parents who completed the survey and were considered eligible were telephoned by our team for additional screening. Finally, parents were interviewed by a clinical psychologist through video conferencing. From each family, participation was limited to one parent, but inclusion of multiple siblings (ages 5–9) was allowed. For families with more than one sibling included, the child identified by the parent as the most anxious was designated as the ‘focus child’. To target the child at highest risk, parents in the CPBC condition were asked to prioritize the focus child when practising parenting strategies. To minimize participant burden, clinician‐led diagnostic interviews with the parents were conducted only for the focus child. By prioritizing diagnostic assessment with the sibling with the highest level of anxiety symptoms, we aimed to reduce the risk of overlooking children with clinical anxiety, ensuring referral for treatment if needed. Before enrollment and at the 12‐month assessment, psychiatric symptoms in the focus child were assessed by clinicians using the ADIS‐C. In the ADIS‐C, the evaluator completes a clinical severity rating (CSR) for each anxiety disorder reflecting severity and interference with functioning. The CSR includes nine ordered scale points (0–8), where a rating of 4 or higher denotes that diagnostic criteria are met. Parental psychiatric symptoms at baseline were assessed using the Mini‐International Neuropsychiatric Interview (M.I.N.I. 7.0.0; Sheehan et al., 1998). In this study, evaluators completed CSRs for parental anxiety based on the M.I.N.I.

Inclusion criteria were (a) the parent suffers from exaggerated anxiety (CSR ≥ 3), (b) the parent speaks and reads Swedish, and (c) the child is 5–9 years and has subclinical anxiety symptoms (CSR of 1–3 for at least one anxiety disorder). The exclusion criteria were as follows: (a) current or recent parental alcohol or substance abuse, (b) severe parental psychiatric conditions (e.g. current or recent psychotic or manic/hypomanic symptoms, severe depression or increased suicide risk), (c) social conditions preventing participation (e.g. ongoing custody dispute, domestic violence, investigation of child neglect through social services), (d) the child has ongoing treatment for anxiety or depression, and (e) the child has no symptoms of anxiety (CSR = 0) or the child meets criteria for depression or an anxiety disorder (CSR ≥4).

During the telephone interview, parents received study information and had the opportunity to ask questions. Written study information, consent forms, and an information pamphlet for children were mailed. The children were also considered study participants, since personal data were collected about them via their parents. Participating parents provided written consent for themselves and their children, and nonparticipating legal guardians provided written consent for the children. Child verbal assent was obtained in a video meeting, where a researcher explained the study, encouraged questions, and asked about willingness to participate. The children did not participate in any other aspect of the study; they did not complete questionnaires, undergo interviews or attend group meetings.

### Procedure

Clinical psychologists conducted video conferencing assessments before enrollment and at the 12‐month assessment. Online questionnaires were administered at baseline, postintervention, and the 12‐month assessment via a secure platform. Data for focus children came from clinician assessments and parent questionnaires, while for siblings, only parent questionnaires were used.

Several safety procedures were implemented. Families with children meeting criteria for an anxiety disorder at baseline were excluded and guided on how to access treatment. In high‐risk situations (e.g. family violence), we guided participants to relevant services and reported to social services when necessary. At follow‐ups, deterioration was monitored by clinical psychologists, offering guidance on how to access support. There were no restrictions on accessing psychiatric care outside the study.

The intervention was administered open label. However, 12‐month evaluators were masked to participant allocation, and participants were instructed not to disclose it. Assessors were instructed to report disclosures. In such cases, a new evaluator should rate an audio recording with the disclosure erased. Data were analyzed by the first and last author, not masked to group allocation.

**Figure 1:**
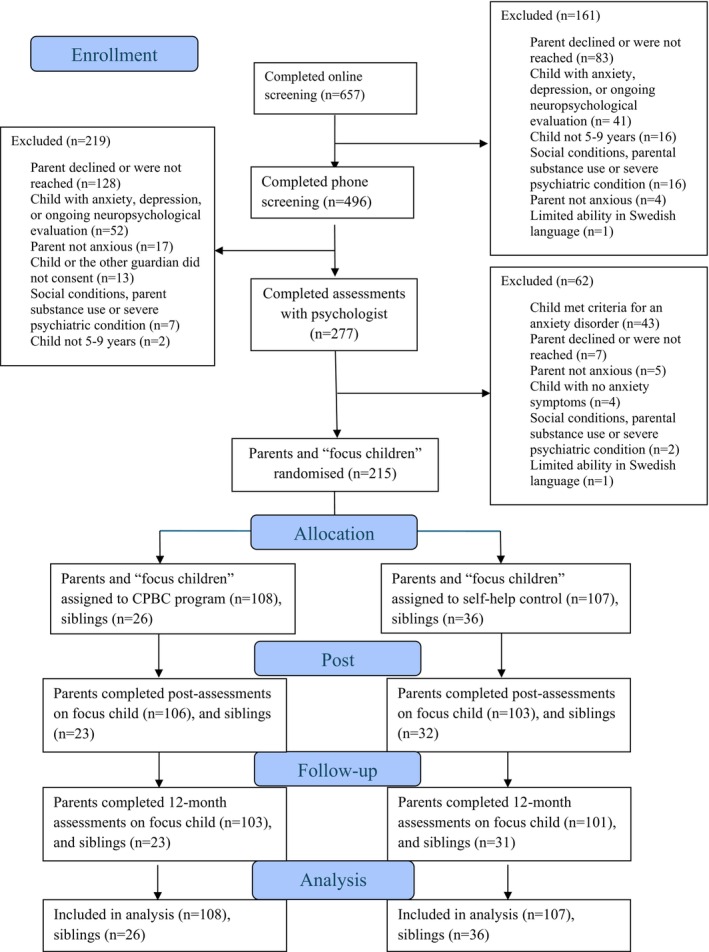
Flow of participants through each stage of the trial

Recruitment took place January 2021–October 2022. Twelve‐month interviews were conducted May 2022–December 2023 by seven assessors (see Table S1). See Figure 1 for flow of participants through the study.

### Randomization

Enrollment and assignment of identification numbers (IDs) were done by research team members. After the baseline assessment, parents were randomized (1:1) to the CPBC or the self‐help control. An external researcher, uninvolved in the study, used a permuted block technique for randomization. To maintain concealment from researchers and evaluators, the random sequence was generated after participant inclusion. After every eight participants were enrolled, a list of study IDs was provided to the external researcher. Using a true random number service (http://www.random.org), the external researcher generated a random list comprising four ‘CPBC’ and four ‘Control’ assignments, merged it with the provided identification list in a time‐stamped spreadsheet and returned it to the research team.

### Interventions

**Figure 2:**
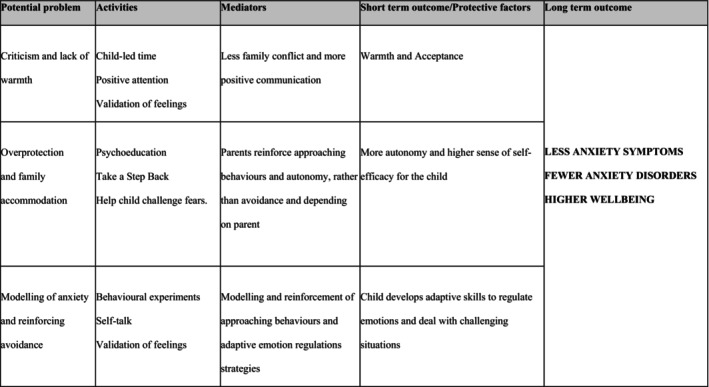
Logic model of the confident parents–Brave Children Program

The CPBC was developed by the first, second, and last author. It includes six 120‐min group sessions over 10 weeks, and 4 weeks after the last group session, an individual 30‐min booster. All sessions were conducted through video conferencing. Each group consisted of four parents and was led by a clinical psychologist. Parents received a workbook containing psychoeducational texts and worksheets. The session structure was outlined in the therapist manual. During sessions, the group leader followed up on and supported planning of home assignments, presented the present topic, and facilitated discussions. Parenting skills addressed in the CPBC are positive reinforcement, child‐led time, emotional validation, decreasing anxious modeling and overprotection, and strategies to help children overcome anxiety. Figure 2 presents a logic model of the CPBC program. For session content, see Table S2.

The active comparator in this study was a Swedish parenting book, containing positive and effective parenting strategies (Lalouni & Lönn Rhodin, 2019). Control parents were instructed to read the book and implement the described strategies over 10 weeks, with no support from the research team. The content of the book is summarized in Table S3.

### Measures

#### Primary outcome

For Research Questions 1 and 2, we used our primary outcome measure, the ADIS‐C (Lyneham & Rapee, 2005). The ADIS‐C has undergone rigorous validation procedures (Wood, Piacentini, Bergman, McCracken, & Barrios, 2002). In this study, the ADIS‐C was solely administered to parents. Evaluators were clinical psychologists who received training on ADIS‐C and completed joint ratings to assess inter‐rater reliability each semester. We evaluated change in CSRs (as an ordinal outcome) and diagnostic status (as a binary outcome).

#### Secondary outcomes

To address Research Question 3, we used the Screen for Child Anxiety‐Related Disorders—Revised (SCARED‐41; Ivarsson, Skarphedinsson, Andersson, & Jarbin, 2018). For the fourth research question, we used the Parenting Sense of Competence Scale (Gilmore & Cuskelly, 2009).

Several additional questionnaires, not linked to the research questions of this report, were administered. Relevant parenting behaviors were assessed in alignment with preregistered Research Question 5 on mediation analysis. Further assessments were conducted to evaluate parental symptoms of anxiety and depression, which were not related to the preregistered research questions. Satisfaction was measured postintervention using The Client Satisfaction Questionnaire‐8 (CSQ‐8; Attkisson & Zwick, 1982).

Details on the secondary measures are presented in Appendix 1. The timing for all study measurements is available in Table S4.

#### Process outcomes

Advanced psychology master students assessed the group leader's adherence to the treatment manual by listening to a stratified random sample of audio recordings, covering 20% of the sessions. An adapted version of the Body Project Session Adherence (Ghaderi, Stice, Andersson, Persson, & Allzén, 2020) was used, with ratings performed on a 10‐point scale, ranging from 0 (no adherence) to 10 (perfect adherence).

For CPBC parents, the number of attended sessions was used to measure treatment adherence. To measure treatment engagement, group leaders rated homework completion for each participant and session, on a 5‐point scale (1, not done; 2, a small part done; 3, partially done; 4, done; and 5, done and more). Additionally, participants were asked about the frequency of using the CPBC skills. For control participants, compliance was measured by asking how much of the book they had read (none, a quarter, half, three‐quarters, or all), and engagement by how often they used the parenting skills from the book.

### Statistical analysis

To determine sample size, we utilized findings from the trial by Ginsburg et al. (2015). A sample size of 194 was calculated to have an 80% power to detect an effect equivalent to an odds ratio of 0.33 (with an expected prevalence of 25% anxiety disorders in the control group at the 12‐month assessment) or a standardized mean difference (Cohen's *d*) of 0.40. This estimation considered a conservative approach, reflecting less than half of the expected effect observed in the CAPS study. Anticipating a 10% attrition rate, we aimed at recruiting 216 parents.

We used an intention‐to‐treat approach, including all available data in all analyses, with no cases omitted. We coded time as a categorical variable to allow for flexibility in modeling nonlinear trends between time points. To assess the primary outcome, we investigated change in CSRs using a cumulative link mixed model (CLMM), with a random intercept to account for the nested design (assessments nested within participants). The coefficients of the predictors in a CLMM represent the log odds for moving to a higher CSR category. We explored between‐group differences in prevalence of anxiety disorders (CSR ≥ 4) at the 12‐month assessment using logistic regression.

To investigate potential moderators of the intervention effect, we examined parental anxiety severity (measured by the highest CSR for an anxiety disorder), child gender (girls vs. boys), and child age and anxiety symptoms (SCARED‐41 scores) pretreatment. In CLMMs, we explored three‐way interactions (condition*time*moderator) to identify differences across moderators (Hesser, 2015). In cases of a statistically significant three‐way interaction, we fitted separate CLMMs for dichotomized categories of the moderator to facilitate the interpretation of the effects.

For Research Questions 3 and 4, we employed linear random effects regressions across all three time points. We incorporated random intercepts to account for the nested design (participants nested within sibling pairs for Research Question 3, and assessments nested within participants for both research questions).

For inter‐rater reliability, we calculated the Krippendorff's alpha, suitable for nominal and ordinal data and when different evaluators have rated different sets of cases. Values above 0.67 and 0.80 are considered acceptable and good, respectively (Hayes & Krippendorff, 2007). Within‐group and between‐group effect sizes for continuous outcomes (i.e. Cohen's *d*) were calculated using model‐based estimates derived from the coefficients for the slope (or slope difference), divided by the baseline standard deviation, as recommended for repeated measures models (Feingold, 2009). We used 95% confidence intervals to explore statistical significance. Statistical analyses were performed in the R software (R Core Team, 2022) using the ‘lmerTest’, ‘ordinal’, and the ‘irr’ packages.

## Results

A total of 215 parents and 277 children were included. For sociodemographic and clinical characteristics of the participants, see Table 1. The postassessment was completed by 209 parents (97.2%) and the 12‐month assessment by 204 parents (94.9%). The inter‐rater reliability between assessors for CSRs (ordinal data) and anxiety disorders (nominal data) was high, Krippendorff's alpha = 0.93 and 0.82, respectively.

**Table 1:** Baseline demographics and clinical characteristics

	CPBC		Active control	
	*n* = 108 parents		*n* = 107 parents	
	*n* = 134 children		*n* = 143 children	
	*n* (%)	*M* (*SD*)	*n* (%)	*M* (*SD*)
Age (focus child)		7.0 (1.4)		7.2 (1.4)
Age (sibling)		6.7 (1.5)		6.4 (1.2)
Gender (focus child)				
Girls	54 (50%)		52 (49%)	
Boys	54 (50%)		55 (51%)	
Gender (sibling)				
Girls	11 (42%)		16 (44%)	
Boys	15 (58%)		20 (56%)	
Highest CSR (focus child)		2.5 (0.7)		2.5 (0.6)
Age (parent)		38.8 (4.7)		39.0 (3.9)
Birth country (parent)				
Sweden	95 (88%)		97 (91%)	
Other European country	9 (8%)		6 (6%)	
Outside Europe	4 (4%)		3 (3%)	
Educational level (parent)				
≤12 years	3 (3%)		11 (10%)	
University studies <3 years	15 (14%)		19 (18%)	
University studies ≥3 years	90 (83%)		77 (72%)	
Current care mental health (parent)	47 (44%)		43 (40%)	
Current therapy (parent)	16 (15%)		19 (18%)	
Previous therapy (parent)	88 (81%)		87 (81%)	
Current psychotropics (parent)	49 (45%)		44 (41%)	
CSR parent principal anxiety disorder		4.3 (0.9)		4.2 (0.9)
Anxiety disorders (parent)^a^				
GAD	67 (62%)		66 (62%)	
Panic disorder	13 (12%)		10 (9%)	
Agoraphobia	9 (8%)		11 (10%)	
Health anxiety	7 (6%)		5 (5%)	
Social anxiety disorder	13 (12%)		14 (13%)	
Specific phobia	18 (17%)		10 (9%)	
No anxiety disorder^b^	25 (23%)		27 (25%)	
Major depressive disorder (parent)	7 (6%)		10 (9%)	

CPBC, Confident Parents–Brave Children; CSR, Clinical Severity Rating; GAD, Generalized anxiety disorder.

^a^ Some participants met criteria for multiple anxiety disorders.

^b^ CSR = 3.

Regarding Research Question 1, no statistically significant difference was found between CPBC children and control children regarding moving to a higher CSR category from baseline to the 12‐month assessment, OR = 0.67 (95% CI: 0.31, 1.49). Further, we did not find a statistically significantly lower prevalence of anxiety disorder at the 12‐month assessment in the CPBC compared to the control condition, OR = 0.57 (95% CI: 0.24, 1.31). Change in CSRs is presented in Table 2, and specified anxiety disorders divided by group are presented in Table S5.

**Table 2:** Changes in Primary Outcome (CSR) between pre‐ and 12‐months assessment divided by condition

	CPBC (*n* = 108)		Active control (*n* = 107)	
	*N*	%	*N*	%
Disordered	10	9.3	16	15.0
Deteriorated	16	14.8	16	15.0
No change	53	49.1	47	43.9
Improved	24	22.2	22	20.6
Missing	5	4.6	6	5.6

CPBC, Confident Parents–Brave Children; CSR, Clinical Severity Rating (in AnxietyDisorders Interview Schedule—Schedule for Children). Disordered = Increased CSR to a 4 or above, Deteriorated = Increased CSR but below 4, No change = Same CSR at both assessments, Improved = Decreased CSR.

Regarding Research Question 2, the moderation analyses demonstrated a statistically significant three‐way interaction regarding child age (condition*time*age), OR = 1.81 (95% CI: 1.01, 3.27), that is the intervention effect varied with age. We divided children into two groups based on the median age. In separate analyses of 5–6‐year‐olds, CPBC showed lower risk of increased CSR, OR = 0.24 (95% CI: 0.08, 0.77), and anxiety diagnosis, OR = 0.23 (95% CI: 0.05, 0.84), compared to control children. Among 7–9‐year‐olds, no statistically significant differences were found between CPBC and control children regarding risk of increased CSR, OR = 1.77 (95% CI: 0.57, 5.50), or anxiety diagnosis, OR = 1.21 (95% CI: 0.38, 3.81). No other moderator showed statistically significant three‐way interactions. Table S6 shows changes in CSRs and anxiety diagnosis by age group. Table S7 displays the statistical results from all moderation analysis.

Regarding Research Question 3, we found a statistically significant effect, where CPBC children showed larger decreases in anxiety symptoms between baseline and the 12‐month assessment compared to control children, β = −3.38 (95% CI: −5.31, −1.45). No statistically significant effect was seen between pre‐ and postassessment, β = −1.79 (95% CI: −3.71, 0.12). Means, standard deviations, within‐ and between‐group effect sizes across time points are presented in Table 3.

**Table 3:** Secondary outcome measures at baseline, post and 12‐months

Measure	CPBC (*n* = 108)					Active control (*n* = 107)					CPBC vs. active control	
	Pre	Post	12 m	Effect pre‐post	Effect pre‐12 m	Pre	Post	12 m	Effect pre‐post	Effect pre‐12 m	Between‐group effect pre‐post	Between‐group effect pre‐12 m
	*M* (*SD*)	*M* (*SD*)	*M* (*SD*)	Cohen's *d* (95% CI)	Cohen's *d* (95% CI)	*M* (*SD*)	*M* (*SD*)	*M* (*SD*)	Cohen's *d* (95% CI)	Cohen's *d* (95% CI)	Cohen's *d* (95% CI)	Cohen's *d* (95% CI)
SCARED‐41^a^	16.4 (9.4)	14.8 (9.1)	13.2 (8.9)	**−0.17 (−0.31, −0.02)**	**−0.35 (−0.49, −0.20)**	15.1 (9.7)	15.3 (9.8)	15.0 (10.1)	0.02 (−0.12, 0.16)	0.01 (−0.13, 0.15)	−0.19 (−0.39, 0.01)	**−0.35 (−0.55, −0.15)**
PSOC	42.5 (8.5)	44.8 (8.0)	46.4 (7.9)	**0.27 (0.13, 0.41)**	**0.47 (0.33, 0.61)**	43.1 (8.2)	44.2 (9.1)	45.8 (9.1)	**0.14 (0.00, 0.28)**	**0.32 (0.18, 0.46)**	0.14 (−0.06, 0.33)	0.16 (−0.04, 0.36)
EEAC	51.4 (13.9)	48.7 (11.2)	48.7 (10.5)	**−0.20 (0.33, 0.07)**	**−0.21 (−0.34, −0.08)**	48.7 (12.3)	49.0 (13.1)	48.9 (13.3)	0.02 (−0.10, 0.13)	0.01 (−0.11, 0.13)	**−0.23 (−0.41, −0.06)**	**−0.23 (−0.41, −0.06)**
RPOS	23.4 (7.0)	18.9 (6.9)	17.3 (6.6)	**−0.63 (−0.79, −0.47)**	**−0.86 (−1.02, −0.70)**	23.8 (7.3)	21.6 (8.1)	19.3 (7.9)	**−0.27 (−0.41, −0.13)**	**−0.59 (−0.73, −0.45)**	**−0.34 (−0.56, −0.13)**	**−0.24 (−0.46, −0.03)**
MPAQ‐AA	17.2 (4.0)	16.2 (3.3)	15.9 (3.2)	**−0.24 (−0.41, −0.07)**	**−0.34 (−0.51, −0.17)**	16.6 (4.0)	15.6 (4.1)	15.4 (4.0)	**−0.24 (−0.39, −0.09)**	**−0.32 (−0.47, −0.17)**	0.01 (−0.22, 0.23)	−0.02 (−0.25, 0.21)
MPAQ‐CC	34.4 (4.2)	34.9 (4.0)	35.6 (3.9)	0.11 (−0.05, 0.28)	**0.30 (0.13, 0.47)**	36.1 (4.7)	35.9 (4.5)	36.9 (4.7)	−0.05 (−0.19, 0.09)	**0.16 (0.02, 0.30)**	0.16 (−0.06, 0.37)	0.11 (−0.11, 0.33)
FASA	11.0 (7.9)	10.3 (7.6)	8.9 (8.0)	−0.06 (−0.22, 0.09)	**−0.29 (−0.44, −0.13)**	9.6 (6.7)	9.3 (7.0)	8.5 (6.6)	−0.05 (−0.21, 0.11)	**−0.17 (−0.33, −0.01)**	−0.02 (−0.24, 0.20)	−0.16 (−0.38, 0.06)
PROMIS	25.0 (5.6)	22.7 (5.8)	22.4 (6.0)	**−0.39 (−0.58, −0.19)**	**−0.44 (−0.64, −0.24)**	24.9 (5.4)	23.4 (6.1)	23.2 (6.3)	**−0.29 (−0.48, −0.10)**	**−0.32 (−0.51, −0.13)**	−0.11 (−0.39, 0.16)	−0.14 (−0.41, 0.14)
PHQ‐9	6.3 (4.7)	6.0 (4.9)	5.9 (5.1)	−0.04 (−0.24, 0.15)	−0.08 (−0.27, 0.12)	6.2 (4.4)	6.4 (5.0)	6.0 (5.2)	0.08 (−0.10, 0.25)	−0.03 (−0.21, 0.15)	−0.11 (−0.38, 0.15)	−0.05 (−0.31, 0.22)

CPBC, Confident Parents–Brave Children; SCARED‐R, Screen for Child Anxiety‐Related Disorders—Revised; PSOC, Parenting Sense of Competence Scale; EEAC, Expressed Emotion Adjective Checklist (Klaus 2011); RPOS, Revised Parental Overprotective Scale Clarke (Clarke, Cooper, & Creswell, 2013); MPAQ‐AA, Modeling of Parental Anxiety Questionnaire – Displaying Anxiety and Avoidance (Elfström & Ahlen, 2022); MPAQ‐CC, Modeling of Parental Anxiety Questionnaire – Being Curious and Content (Elfström & Ahlen, 2022); FASA, Family Accommodation Scale‐Anxiety (Lebowitz et al., 2013); PROMIS, Patient‐Reported. Outcomes Measurement Information System (Pilkonis et al. 2011), PHQ9, Patient Health Questionnaire‐9 (Kroenke et al. 2001). Statistically significant results in bold.

^a^ Including siblings (i.e. *N* = 277).

Concerning Research Question 4, there was no statistically significant difference between conditions between pre‐ and postassessment, β = 1.13 (95% CI: −0.51, 2.76), or between pre‐ and the 12‐month assessment, β = 1.35 (95% CI: −0.30, 3.00). According to within‐group effect sizes, CPBC parents showed increased parental self‐efficacy at post and at 12‐month assessment, while control parents showed increases only at the 12‐month assessment. Means, standard deviations, and within‐ and between‐group effect sizes across time points are presented in Table 3. See Table S7 for detailed results of all regression analyses.

Regarding the secondary outcomes not related to this report's research questions, means, standard deviations, and effect sizes (Cohen's *d*) are presented in Table 3. According to the between‐group comparisons, CPBC parents showed larger decreases in criticism and overprotection compared with control parents. No significant between‐group effect sizes were observed for anxious modeling, family accommodation, or parental symptoms of anxiety or depression.

Ratings of group leaders' adherence to the manual ranged from 6 to 10, with a mean of 9.63 (*SD* = 0.81), indicating high adherence. The mean number of attended group sessions was 5.01 (*SD* = 1.64) and 90% participated in three or more of the six group sessions. Further, 80% attended the individual booster. The mean homework completion score across sessions was 3.50 (*SD* = 1.41) on a scale from 1 to 5. In the control group, 45% read the entire book, 15% three quarters, 13% half, 10% a quarter, and 13% none (4% did not respond). At the 12‐month assessment, 52% of CPBC parents and 16% of control parents reported using strategies learned weekly. The mean CSQ‐8 score was 28.34 (*SD* = 3.55) in the CPBC condition and 24.66 (*SD* = 4.77) in control condition.

## Discussion

The current study aimed to assess the efficacy of a program for anxious parents, the CPBC, in preventing child anxiety disorders and symptoms. Compared with a self‐help control, no significant effect on child CSR or diagnostic status was found at the 12‐month assessment. However, when stratifying by age, children 5–6 years in CPBC showed lower risk of increased CSR and anxiety diagnosis, compared with controls. Further, a significant between‐group effect was found on child anxiety symptoms, as measured by the SCARED‐41 at the 12‐month assessment. Increased parental self‐efficacy was seen for CPBC parents both at the post‐ and the 12‐month assessment, but for control parents only at the 12‐month assessment.

Over the 12‐month follow‐up period, CPBC parents reported a significant reduction in their children's anxiety symptoms, with SCARED‐41 scores decreasing from 16.4 to 13.2. The control group's scores remained unchanged (15.1 to 15.0). To put these results into context, it can be mentioned that Behrens, Swetlitz, Pine, and Pagliaccio (2019) found that a large sample of healthy children had a mean SCARED‐41 score of 12.1, whereas a large sample of children with anxiety disorders had a mean score of 18.9. The effect found on SCARED‐41 in the current study should be interpreted in the context of prevention research, where smaller effect sizes are anticipated compared to treatment research (Ishikawa, Okajima, Matsuoka, & Sakano, 2007; Werner‐Seidler et al., 2021).

The lack of a significant effect on the primary outcome across the entire group of children suggests that the CPBC did not achieve its goal of preventing anxiety disorders. This warrants consideration from several perspectives. First, it highlights the need for a critical evaluation of the intervention's underlying logic model. The logic model states that (a) parental anxiety predicts maladaptive parenting, (b) our intervention improves parenting behaviors, and (c) these parenting behaviors affect child anxiety. Weak associations at any link in this chain can reduce the intervention's effectiveness. Mediation analysis, one approach to testing the logic model's validity, is beyond this paper's scope but will be addressed in a separate publication. Second, in this study, we may have partially targeted an age group of children for whom the intervention was not suitable. Our results indicate that the CPBC was effective in preventing anxiety disorders only in 5–6‐year‐olds, possibly indicating that the underlying logic model is more applicable for young children. Parent‐only programs might be more potent in early childhood when parents are highly involved in most aspects of the child's life. It is possible that 7–9‐year‐olds would benefit more from a prevention program that not only involves the parents but also directly engages the children, providing them with strategies to manage anxiety independently. Third, although power calculations were based on adjacent research, this study turned out to be underpowered. When calculating power we relied on the CAPS study (Ginsburg et al., 2015), expecting 25% of control children to meet criteria for an anxiety disorder by the 12‐month assessment. However, at the 12‐month assessment, only 15% of the control children in the current trial had developed an anxiety disorder. This lower rate could indicate that we failed to identify families with children in a well‐defined high‐risk group.

Although the evidence base for anxiety prevention is limited, existing research suggests that selective or indicated approaches are more effective than universal strategies (Werner‐Seidler et al., 2021). This trial aligns with selective prevention principles by focusing on children of anxious parents, but it also incorporates some elements of indicated prevention, as children with very low anxiety levels (CSR 0 on all anxiety disorders) were excluded. Given the lower‐than‐expected rate of anxiety disorders in control children, future work could enhance the precision of high‐risk group identification. For example, interventions could include only families where parents meet diagnostic criteria for anxiety disorders or demonstrate specific problematic parenting behaviors, such as overprotection.

It is premature to draw definitive conclusions about the effectiveness of preventive interventions targeting anxious parents. Prior to this trial, only one other full‐scale RCT specifically designed as a true prevention study, with the occurrence of anxiety disorders in children as the primary outcome, had been published. In this preceding RCT, Ginsburg et al. (2015) evaluated the family intervention CAPS. When comparing the results from the two trials, the CPBC appears clearly less effective than the CAPS. However, there are a few factors that are relevant to highlight when interpreting these results. First, the difference in effect may in part be explained by the fact that fewer control children in the current trial developed an anxiety disorder after 12 months compared with Ginsburg's trial (15% vs. 31%). Second, Ginsburg employed a passive control condition, in contrast to the current study, where control parents received a parenting book. Both control and CPBC parents reported high levels of satisfaction and compliance. The content of the CPBC and the control condition parenting book differ significantly: only the CPBC includes strategies to reduce overprotection and anxious modeling and to support children in overcoming fears. However, positive parenting strategies, including child‐led time and positive reinforcement, are presented in the CPBC program and the control condition parenting book. Although all within‐group effects should be interpreted cautiously, it is notable that increases in parental self‐efficacy were seen in both conditions. Hence, it should be considered that these two factors could account for part of the difference in observed effects in the two trails, rather than solely reflecting a lower efficacy of the CPBC. Furthermore, to positively impact public health, preventive interventions need to be both effective and scalable. While the CAPS requires 11 in‐person therapist hours per family, the CPBC involves 12 h per group (equivalent to three therapist hours per parent) and is delivered online. Though this trial's findings are not conclusive, they suggest the CPBC holds promise for preventing anxiety in young children, warranting further exploration of brief, parent‐only interventions. One limitation of this study is that most enrolled parents identified as female. While the study's advertisement aimed to engage parents regardless of gender, we fell short in recruiting fathers. Concerns about fathers not receiving parenting support have been raised (Bergström, 2017; Wells, 2016), and a task for future research is to focus on anxious fathers. The generalizability of the results is further limited by the highly educated and predominantly Swedish‐born study sample. To ensure more representative samples in future studies, strategies could involve recruiting via clinics in diverse areas and offering the intervention in multiple languages. Another limitation of this trial was the lack of participant blinding, which may increase the risk of bias.

## Conclusions

The CPBC program did not demonstrate overall effectiveness in preventing child anxiety disorders and widespread adoption cannot be recommended at this stage. However, the results partially support the potential of CPBC, with a lower rate of anxiety disorders in younger children and reduced anxiety symptoms across all ages.

## Ethical considerations

This study was approved by the Swedish Ethical Review Authority (Dnr: 2020‐03532, 2020‐05897, 2020‐07002). Written informed consent was obtained from parents for themselves and their children. Verbal assent was collected from children in a video meeting.

## Trial registration

This trial was registered at ClinicalTrials.gov (NCT04722731).

**Key points**

- A risk factor for pediatric anxiety disorder is having an anxious parent.
- This is the second true prevention trial evaluating a program for anxious parents to prevent offspring anxiety.
- The results indicate that the parent group program did not have a significant effect on preventing child anxiety disorders within 12 months, compared to a self‐help control. However, a significant effect was found on parent‐rated child anxiety symptoms.
- When stratifying by age, a significant lower risk for anxiety disorders was found in younger children (5–6 years old).
- The CPBC may have potential for preventing anxiety in young children; however, further research is warranted.

## Supporting information


**Table S1.** Number of clinical assessments at the 12‐month follow‐up divided by rater and condition.
**Table S2.** Summary of the Confident Parents – Brave Children program content.
**Table S3.** Summary of the Self‐help Parenting Book content.
**Table S4.** Overview of measures and time points.
**Table S5.** Specified child anxiety disorders divided by groups.
**Table S6.** Changes in primary outcome (CSR) between pre‐ and 12‐months assessment divided by condition and age.
**Table S7.** Regression results.

## Data Availability

The data that support the findings of this study are available upon request from the corresponding author. These data are not publicly available due to privacy or ethical restrictions.

## Appendix 1

The Screen for Child Anxiety Related Emotional Disorders—Revised (SCARED‐41) is a 41‐item parent‐report questionnaire designed to assess anxiety symptoms in children. It evaluates five anxiety‐related domains: panic/somatic symptoms, generalized anxiety, separation anxiety, social anxiety, and school avoidance. Each item is rated on a 3‐point Likert scale, with higher scores indicating more anxiety. The SCARED‐41 has proven useful for screening anxiety symptoms in a Swedish population of children aged 6–17 years (Ivarsson et al., 2018). In an American sample, the parent‐rated SCARED‐41 performed as well for younger children (from 5 years old) as for older children (Sequeira, Silk, Woods, Kolko, & Lindhiem, 2020).

The Parenting Sense of Competence Scale (PSOC) is a self‐report questionnaire designed to assess parents' perceived competence in their parenting role. The scale includes statements rated on a 6‐point Likert scale, where higher scores indicate a greater perceived sense of competence in parenting (after reversing negatively worded items). Factor analysis has identified three subfactors within the scale: satisfaction, efficacy, and interest (Gilmore & Cuskelly, 2009). While the original scale contains 17 items, this study utilized a shorter version with 11 items, excluding the interest subscale.

The PROMIS Anxiety Short Form is an 8‐item brief self‐report questionnaire designed to assess anxiety symptoms. The short form is part of the Patient‐Reported Outcomes Measurement Information System (PROMIS) a comprehensive initiative designed to assess various aspects of mental and physical health. Respondents rate the frequency of their anxiety symptoms over the past seven days on a 5‐point Likert scale, with higher scores indicating greater levels of anxiety. The PROMIS Anxiety Short Form has demonstrated acceptable performance across diverse populations (Teresi, Ocepek‐Welikson, Kleinman, Ramirez, & Kim, 2016).

The Patient Health Questionnaire‐9 (PHQ‐9) is a nine‐item questionnaire designed to assess severity of depressive symptoms. Items corresponds to diagnostic criteria for major depressive disorder and the respondent rates how often they have experienced specific symptoms, such as low mood, loss of interest, or fatigue the past 2 weeks. Items are scored on a 4‐point Likert scale with higher scores indicating greater severity of depression. The PHQ‐9 is widely used in both clinical and research settings for screening and monitoring of depressive symptoms (Levis et al., 2020).

The Revised Parental Overprotection Scale (RPOS) was developed to measure overprotective parenting behaviors. The original version (POS) consists of 19 items intended to evaluate parenting engagement in behaviors aimed to protect the child from perceived dangers (Clarke et al., 2013). We revised the POS based on a Rasch evaluation, and retained 11 items (i.e., the RPOS). Items are scored on a 5‐point Likert scale with higher scores indicating greater overprotection. In our evaluation in a Swedish sample, the RPOS showed sound psychometric properties and was moderately associated to parental anxiety symptoms (Elfström & Åhlén, 2024).

The Expressed Emotion Adjective Checklist (EEAC) is a questionnaire developed to assess parents' perceptions of their emotional expressions toward their child. The checklist includes a set of positive and negative adjectives, and these are rated on an eight‐point Likert scale, where higher scores (after reversing positive adjectives) indicate more criticism. The EEAC has been found to be a useful measure of expressed emotions, easier to implement than previous observational tests (Klaus, Algorta, Yung, & Fristad, 2015).

The Modelling of Parental Anxiety Questionnaire (MPAQ) is a self‐report measure designed to assess the extent to which parents model anxious or non‐anxious behaviors to their children. This study included the seven‐item subscale *Displaying anxiety and avoidance* and the nine‐item subscale *Being curious and content*. Items are scored on a 5‐point Likert scale. Higher scores on the Anxiety and avoidance subscale indicates a high level of anxious and avoidant modelling, and higher scores on the Curious and content subscale indicates a high level of non‐anxious modelling such as encouraging the child to try new things. The MPAQ may, for example, be a suitable measure to identify mediation effects (Elfström & Ahlen, 2022).

The Family Accommodation Scale‐Anxiety (FASA) is a 13‐item questionnaire developed to assess the degree to which parents modify their behaviors in response to a child's anxiety. This can include actions such as providing reassurance or avoiding anxiety‐provoking situations, to reduce the child's distress. Items are scored on a 5‐point Likert scale with higher scores indicating greater family accommodation. Adequately, the scale score has been found to be associated to child anxiety symptoms (Lebowitz et al., 2013).

The Client Satisfaction Questionnaire‐8 (CSQ‐8) is a widely used, eight‐item brief self‐report measure developed to assess client satisfaction with healthcare and mental health services. The items cover perceptions of the quality and adequacy of the services they received. Each item is rated on a 4‐item Likert scale with higher scores indicating high levels of satisfaction (Attkisson & Zwick, 1982).
