# Supplementary material for: Evaluating a program to prevent anxiety in children of anxious parents: a randomized controlled trial
Source: J Child Psychol Psychiatry. 2025 Mar 12;66(9):1345–56. doi: 10.1111/jcpp.14151 (PMC12350814; doi:10.1111/jcpp.14151)
Supplement: Supplementary file 1 — Table S1. Number of clinical assessments at the 12‐month follow‐up divided by rater and condition. Table S2. Summary of the Confident Parents – Brave Children program content. Table S3. Summary of the Self‐help Parenting Book content. Table S4. Overview of measures and time points. Table S5. Specified child anxiety disorders divided by groups. Table S6. Changes in primary outcome (CSR) between pre‐ and 12‐months assessment divided by condition and age. Table S7. Regression results. [file JCPP-66-1345-s001.docx]

**Table S1**

*Number of clinical assessments at the 12-month follow-up divided by rater and condition*

|  | **Rater 1** | **Rater 2** | **Rater 3** | **Rater 4** | **Rater 5** | **Rater 6** | **Rater 7** |
| --- | --- | --- | --- | --- | --- | --- | --- |
|  | n (%) | n (%) | n (%) | n (%) | n (%) | n (%) | n (%) |
| CPBC | 29 (50%) | 28 (50%) | 12 (55%) | 9 (43%) | 10 (53%) | 9 (53%) | 6 (55%) |
| Active control | 29 (50%) | 28 (50%) | 10 (45%) | 12 (57%) | 9 (47%) | 8 (47%) | 5 (45%) |
| Total | 58 | 56 | 22 | 21 | 19 | 17 | 11 |

Notes: CPBC = Confident Parents – Brave Children

**Table S2**

*Summary of the Confident Parents – Brave Children program content*

| **Session** | **Content** |
| --- | --- |
| 1. Introduction | Psychoeducation about anxiety, covering both the cognitive, behavioural, and physical aspects. Anxiety is discussed as a normal part of parenthood and of the typical development for children. *Child led time* is introduced, meaning that parents spend 10-15 minutes daily, engaging in an activity chosen by the child. The parent is instructed o give the child his/her full attention, follow the child's lead, and provide supportive and positive comments.  *Home assignments*   - Child led-time - Simple functional analysis to examines the causes and consequences of anxious behavior |
| 2. Warmth and acceptance | The importance of validating children’s feelings and how this can be done in practice. Validation is introduced as a way of expressing acceptance and strengthening the relationship, but also as a strategy to support the child’s emotion regulation skills. Positive attention is introduced as a way of affecting the child’s behavior in a positive way and at the same time creating a more positive home environment.  *Home assignments*  At least once a day:   - validate your child’s feelings - letting your children know when they have done well (positive reinforcement) |
| 3. Take a step back | Overprotective behaviors were identified, and a plan was made regarding how to increase the children’s’ autonomy. We discuss how many parents protect their children not only from physical harm, but also from experiencing negative emotions. A key message is that when parents do “too much too soon”, the child can be hindered from developing self-confidence and age-appropriate skills. Furthermore, parents are coached to replace general warnings with clear instructions on how to handle the situation.  Home assignment   - Implement a behavioral change: take a step back or reduce warnings |
| 4. Model learning | It is emphasized that anxiety, avoidance, and safety behaviors can be learned through model learning, but that model learning also can be used to help children develop positive coping skills. “Self-talk” is introduced to communicate calmly to the child (e.g., “I got a bit stressed, but there is nothing we can do about the bus being late. Let’s sit down and you can show me your new game while we wait”). During the session, the parents plan a behavioral experiment to carry out together with the child. The parent should confront a mild fear in a playful manner, and then have a discussion with the child about overcoming fears.  Home assignment   - Using self-talk - Behavioral experiment |
| 5. Anxious children | How to recognize and respond to anxiety in children. A distinction is made between how to respond to the child’s anxiety depending on why the child is feeling anxious (e.g., is the fear related to an actual risk?). Most of the session is devoted to how we can help children to overcome anxiety that is excessive in proportion to the actual risk. A few different strategies are thought to help the child overcome anxiety, including confronting fear through play, graded exposure, and role play to prepare for social situations.  Home assignment   - Talk with your child about his/her fears and how they can be challenged - Help your child confront a fear |
| 6. Sum up and plan ahead | The last session consists of two parts. Firstly, we follow up on how to help children confront fears and discuss how to overcome obstacles that can occur along the way. Secondly, the principles learned during the parenting program are reviewed and the participants plan for how to continue to use the learned strategies during the coming month. |
| Individual Booster Session | The focus of the session is decided based on the needs of the participant. The goal is to help the participant get past any difficulties that have occurred when trying to implement the principles of the program and to make an updated plan for the future. |

**Table S3**

*Summary of the Self-help Parenting Book content*

| **Chapter** | **Content** |
| --- | --- |
| **1.** | Introduction to the Psychological Plate Model and its health benefits. The first part in the model, warmth and love between the parent and child, is described as fundamental for child development. Aspects such as being physically close to the child, being attentive, but also good enough parenting is described. |
| **2.** | The second chapter concerns spending time with the child. Two main types are described: 1) spending time together in the family more generally, and the more specific 2) child-directed time, in which the child leads an activity and the parent focus on being present, commenting and showing enthusiasm. |
| **3.** | The third chapter’s theme is confirmation. It emphasizes that the parent should use more positive than negative feedback. Communication skills, such as listening to the child, confirming difficulties, and asking the child for solutions are also highlighted in the chapter. |
| **4.** | Self-esteem and sympathy are focuses of the fourth chapter. Being kind to others is highlighted, and regulating one’s emotions is described as a process that children have to learn. Confirming and putting words to the child’s feelings are described as key to help the child to self-regulate. |
| **5.** | The fifth chapter concern setting boundaries and how these can be communicated in a way that increases the chance that they are adhered to by the child. Picking one’s battles is introduced as a means to decrease the number of prompts to the child. |
| **6.** | Recovery for parents is the theme of the sixth chapter. Stress is described as a state making parenting more difficult, increasing the risk of saying or doing things that the parent might regret. The benefits of regular recovery are presented, and parental support offered by society is described. |
| **7.** | The last chapter of the book concern how behaviors are learnt and how parents can help children in difficult situations. A model of prompting and reinforcing certain behaviors is presented. The model is applied to difficult situations such as setting boundaries for screen time and conflicts between siblings. |

**Table S4**

*Overview of measures and time points*

| **Timeline**  **Measure** | **Online screening** | **Phone screening** | **Video assessment** | **Base-line questionnaire** | **Post**  **measures** | **12-moth assessment** |
| --- | --- | --- | --- | --- | --- | --- |
| Clinician assessed |  |  |  |  |  |  |
| ADIS-C/P |  |  | X |  |  | X |
| MINI |  |  | X |  |  |  |
| Child CSR |  |  | X |  |  | X |
| Parent CSR |  |  | X |  |  |  |
| Parent reported |  |  |  |  |  |  |
| Demographic information | X |  |  |  |  |  |
| EQ5D |  |  |  | X | X | X |
| CHU9D |  |  |  | X | X | X |
| TIC-P |  |  |  | X | X | X |
| EEAC |  |  |  | X | X | X |
| RPOS |  |  |  | X | X | X |
| MPAQ |  |  |  | X | X | X |
| SCARED-R |  |  |  | X | X | X |
| PHQ-9 |  |  |  | X | X | X |
| PROMIS- Anxiety Short Form |  |  |  | X | X | X |
| FASA |  |  |  | X | X | X |
| PSOC |  |  |  | X | X | X |
| CSQ-8 |  |  |  |  | X |  |

# Notes: ADIS-C/P=The Anxiety and Related Disorders Interview Schedule for Children and Parents (1) , MINI= Mini International Neuropsychiatric Interview(2) , CSR= clinical severity rating, EQ5D_VAS = EuroQol-5 Dimensions visual analogue scale (3), CHU9D = the Child Health Utility instrument (CHU9D) (4), TIC-P= Treatment Inventory of Costs in Patients with psychiatric disorders (5), EEAC = Expressed Emotion Adjective Checklist (6), RPOS = Revised Parental Overprotective Scale Clarke (7), MPAQ = the Modeling of Parental Anxiety Questionnaire (8), SCARED-R= Screen for Child Anxiety Related Emotional Disorders (9), PHQ9 = Patient Health Questionnaire-9 (10), PROMIS = Patient-Reported . Outcomes Measurement Information System (11), FASA = Family Accommodation Scale-Anxiety (12), PSOC= Parenting Sense of Competence Scale (13), CSQ-8=Client Satisfaction Questionnaire-8 (14)

**Table S5**

*Specified child anxiety disorders divided by groups*

| **Childhood anxiety disorder** | **CPBC (*n*=108)** | **Active control (*n*=107)** |
| --- | --- | --- |
|  | *n* (%) | *n* (%) |
| Separation Anxiety Disorder | 1 (0.9%) | 7 (6.5%) |
| Social Anxiety Disorder | 5 (4.6%) | 4 (3.7%) |
| Specific Phobia | 5 (4.6%) | 4 (3.7%) |
| Generalized Anxiety Disorder | 1 (0.9%) | 4 (3.7%) |

**Table S6**

*Changes in primary outcome (CSR) between pre- and 12-months assessment divided by condition and age*

|  | **Age 5-6** | | | | **Age 7-9** | | | |
| --- | --- | --- | --- | --- | --- | --- | --- | --- |
|  | **CPBC (n=58)** | | **Active control (n=50)** | | **CPBC (n=50)** | | **Active control (n=57)** | |
|  | *N* | *%* | *N* | *%* | *N* | *%* | *N* | *N* |
| Disordered | 3 | 5.2% | 9 | 18.0% | 7 | 14.0% | 7 | 12.3% |
| Deteriorated | 9 | 15.5% | 8 | 16.0% | 7 | 14.0% | 8 | 14.0% |
| No change | 28 | 48.3% | 24 | 48.0% | 25 | 50.0% | 23 | 40.4% |
| Improved | 17 | 29.3% | 6 | 12.0% | 7 | 14.0% | 16 | 28.1% |
| Missing | 1 | 1.7% | 3 | 6.0% | 4 | 8.0% | 3 | 5.3% |

Notes: CPBC = Confident Parents – Brave Children, CSR = Clinical Severity Rating (in Anxiety Disorders

Interview Schedule - Schedule for Children). Disordered = Increased CSR to a 4 or above,

Deteriorated = Increased CSR but below 4, No change = Same CSR at both assessments, Improved = Decreased CSR.

**Table S7***Regression results*

| **RQ** | **Outcome** | **Fixed effects** | **Estimate** | **SE** | **p-value** |
| --- | --- | --- | --- | --- | --- |
| 1 | ADIS - highest CSR | CPBC | -0.05 | 0.34 | 0.881 |
|  |  | 12 month assessment | 0.42 | 0.29 | 0.148 |
|  |  | CPBC*12 months assessment | -0.39 | 0.40 | 0.329 |
|  |  |  |  |  |  |
| 1 | ADIS - anxiety disorder | Intercept | -1.67 | 0.27 | <0.001*** |
|  |  | CPBC | -0.56 | 0.43 | 0.193 |
|  |  |  |  |  |  |
| 2a | ADIS - highest CSR | CPBC | 0.63 | 0.54 | 0.248 |
|  | (Mod=Age) | 12 month assessment | 0.89 | 0.48 | 0.066 |
|  |  | Age | 0.29 | 0.18 | 0.115 |
|  |  | CPBC*12 months assessment | -1.35 | 0.64 | 0.035* |
|  |  | CPBC*Age | -0.40 | 0.25 | 0.119 |
|  |  | 12 months assessment* Age | -0.26 | 0.22 | 0.230 |
|  |  | CPBC*12 months assessment* Age | 0.59 | 0.30 | 0.049* |
|  |  |  |  |  |  |
| 2b | ADIS - highest CSR | CPBC | 0.29 | 0.47 | 0.535 |
|  | (Mod=Gender) | 12 month assessment | 0.36 | 0.40 | 0.368 |
|  |  | Girls | 0.24 | 0.48 | 0.624 |
|  |  | CPBC*12 months assessment | -0.81 | 0.56 | 0.151 |
|  |  | CPBC*Girls | -0.72 | 0.68 | 0.289 |
|  |  | 12 months assessment*Girls | 0.13 | 0.57 | 0.822 |
|  |  | CPBC*12 months assessment*Girls | 0.87 | 0.81 | 0.283 |
|  |  |  |  |  |  |
| 2c | ADIS - highest CSR | CPBC | -0.49 | 0.55 | 0.375 |
|  | (Mod=Parent CSR) | 12 month assessment | 0.35 | 0.45 | 0.438 |
|  |  | CSR | -0.16 | 0.26 | 0.552 |
|  |  | CPBC*12 months assessment | -0.43 | 0.66 | 0.515 |
|  |  | CPBC*CSR | 0.36 | 0.37 | 0.319 |
|  |  | 12 months assessment* CSR | 0.06 | 0.31 | 0.852 |
|  |  | CPBC*12 months assessment* CSR | 0.02 | 0.43 | 0.958 |
|  |  |  |  |  |  |
| 2d | ADIS - highest CSR | CPBC | -0.21 | 0.61 | 0.731 |
|  | (Mod= SCARED) | 12 month assessment | 0.19 | 0.55 | 0.730 |
|  |  | SCARED | 0.10 | 0.02 | <0.001*** |
|  |  | CPBC*12 months assessment | -0.16 | 0.80 | 0.844 |
|  |  | CPBC*SCARED | 0.00 | 0.03 | 0.876 |
|  |  | 12 months assessment* SCARED | 0.02 | 0.03 | 0.549 |
|  |  | CPBC*12 months assessment* SCARED | -0.02 | 0.04 | 0.660 |
|  |  |  |  |  |  |
| 3 | SCARED - anxiety | Intercept | 15.09 | 0.82 | <0.001*** |
|  |  | CPBC | 1.28 | 1.18 | 0.280 |
|  |  | post assessment | 0.22 | 0.68 | 0.747 |
|  |  | 12 month assessment | 0.10 | 0.69 | 0.887 |
|  |  | CPBC*post assessment | -1.79 | 0.98 | 0.068 |
|  |  | CPBC*12 months assessment | -3.38 | 0.99 | <0.001*** |
|  |  |  |  |  |  |
| 4 | PSOC – self-efficacy | Intercept | 43.08 | 0.82 | <0.001*** |
|  |  | CPBC | -0.62 | 1.15 | 0.597 |
|  |  | post assessment | 1.18 | 0.60 | 0.049* |
|  |  | 12 month assessment | 2.62 | 0.60 | <0.001*** |
|  |  | CPBC*post assessment | 1.13 | 0.84 | 0.180 |
|  |  | CPBC*12 months assessment | 1.35 | 0.85 | 0.111 |

Notes: RQ=Research Question, ADIS=The Anxiety and Related Disorders Interview Schedule for Children and Parents (1), SCARED= Screen for Child Anxiety Related Emotional Disorders (9), PSOC= Parenting Sense of Competence Scale (13), CPBC= Confident Parents- Brave Children

**References**

1. Wood JJ, Piacentini JC, Bergman RL, McCracken J, Barrios V. Concurrent Validity of the Anxiety Disorders Section of the Anxiety Disorders Interview Schedule for DSM-IV: Child and Parent Versions. Journal of Clinical Child and Adolescent Psychology. 2002;31(3):335–42.

2. Sheehan. The Mini-International Neuropsychiatric Interview (M.I.N.I.): The Development and Validation of a Structured Diagnostic Psychiatric Interview for DSM-IV and ICD-10. Vol. 59, J Clin Psychiatry. 1998.

3. König HH, Born A, Günther O, Matschinger H, Heinrich S, Riedel-Heller SG, et al. Validity and responsiveness of the EQ-5D in assessing and valuing health status in patients with anxiety disorders. Health Qual Life Outcomes. 2010 May 5;8:47.

4. Furber G, Segal L. The validity of the Child Health Utility instrument (CHU9D) as a routine outcome measure for use in child and adolescent mental health services. Health Qual Life Outcomes. 2015;13(1):1–14.

5. Bouwmans C, De Jong K, Timman R, Zijlstra-Vlasveld M, Van Der Feltz-Cornelis C, Tan SS, et al. Feasibility, reliability and validity of a questionnaire on healthcare consumption and productivity loss in patients with a psychiatric disorder (TiC-P). BMC Health Serv Res [Internet]. 2013 Dec 15 [cited 2020 Jun 3];13(1):217. Available from: https://bmchealthservres.biomedcentral.com/articles/10.1186/1472-6963-13-217

6. Klaus Nicole m. Validity of the Expressed Emotion Adjective Checklist (EEAC) in Caregivers of Children with Mood Disorders. Bone [Internet]. 2011;23(1):1–7. Available from: https://www.ncbi.nlm.nih.gov/pmc/articles/PMC3624763/pdf/nihms412728.pdf

7. Clarke K, Cooper P, Creswell C. The Parental Overprotection Scale: Associations with child and parental anxiety. J Affect Disord [Internet]. 2013 Nov;151(2):618–24. Available from: http://proxy.kib.ki.se/login?url=https://search.ebscohost.com/login.aspx?direct=true&db=psyh&AN=2013-27815-001&site=ehost-live

8. Elfström S, Ahlen J. Development and validation of the Modeling of Parental Anxiety Questionnaire. J Anxiety Disord. 2022;85(August 2021).

9. Ivarsson T, Skarphedinsson G, Andersson M, Jarbin H. The Validity of the Screen for Child Anxiety Related Emotional Disorders Revised (SCARED-R) Scale and Sub-Scales in Swedish Youth. Child Psychiatry Hum Dev. 2018;49(2):234–43.

10. Vinet L, Zhedanov A. The PHQ-9： Validity of a Brief Depression Severity Measure Kurt. J Phys A Math Theor. 2011;44(8):606–13.

11. Pilkonis PA, Choi SW, Reise SP, Stover AM, Riley WT, Cella D. Item Banks for Measuring Emotional Distress From the Patient-Reported Outcomes Measurement Information System (PROMIS®): Depression, Anxiety, and Anger. 2011 [cited 2021 Apr 23]; Available from: www.nihpromis.org

12. Lebowitz ER, Woolston J, Bar-Haim Y, Calvocoressi L, Dauser C, Warnick E, et al. Family accommodation in pediatric anxiety disorders. Depress Anxiety. 2013;30(1):47–54.

13. Gilmore L, Cuskelly M. Factor structure of the Parenting Sense of Competence scale using a normative sample. Child Care Health Dev. 2009;35(1):48–55.

14. Attkisson CCI, Zwick R. THE CLIENT SATISFACTION QUESTIONNAIRE Psychometric Properties and Correlations with Service Utilization and Psychotherapy Outcome. Eveluation and Program Planning. 1982;5:233–7.
